# PML regulates neuroprotective innate immunity and neuroblast commitment in a hypoxic–ischemic encephalopathy model

**DOI:** 10.1038/cddis.2016.223

**Published:** 2016-07-28

**Authors:** Vuk Palibrk, Rajikala Suganthan, Katja Scheffler, Wei Wang, Magnar Bjørås, Stig Ove Bøe

**Affiliations:** 1 Department of Medical Biochemistry, Oslo University Hospital and University of Oslo, Oslo, Norway; 2 Institute for Cancer Research and Molecular Medicine, Norwegian University of Science and Technology, Trondheim, Norway; 3 Department of Microbiology, Oslo University Hospital, University of Oslo, Oslo, Norway

## Abstract

Regulation of innate immune responses and activation of tissue regenerative processes are key elements in the pathophysiology of brain injuries. The promyelocytic leukemia (PML) gene was originally identified on a breakpoint of chromosomal translocation t(15;17) associated with acute PML. We have studied the role of PML protein during acute and regenerative phases after hypoxia–ischemia (HI) in brains of neonatal mice. We found that PML prevents tissue loss and apoptotic cell death selectively in subcortical regions of the brain at early stages after damage. In accordance with this, we revealed that PML is important for microglia activation and production of key inflammatory cytokines such as IL1*α*, IL1*β*, IL1RN, CXCL10, CCL12 and TNF*α*. During the regenerative phase, PML-depleted mice were found to have impaired transformation of transit-amplifying precursors into migratory progenitors. This was accompanied by increased ratios of symmetric *versus* asymmetric neural progenitor cell divisions during tissue repair and a specific defect in tissue restoration within the striatum 42 days after HI. The data demonstrate a dual role of PML in protection and recovery after brain injury.

The promyelocytic leukemia (PML) protein is a tumor suppressor, which is involved in the t(15;17) chromosomal translocation associated with acute promyelocytic leukemia (APL).^1^ In APL, the t(15;17) chromosomal translocation juxtaposes PML gene to the gene encoding retinoic acid receptor-*α* (RARA). The PML/RARA, a protein product of this chromosomal translocation, supports malignant transformation and growth of hematopoietic precursor cells at the promyelocytic stage of differentiation.^2, 3, 4^ A hallmark of PML is its ability to organize the formation of chromatin-associated nuclear structures called PML bodies. These bodies can be detected by microscopy as numerous doughnut-shaped compartments within the nucleus.^5^ PML has been shown to function in several biological processes, including apoptosis,^6^ genome maintenance,^7, 8^ senescence,^9, 10^ proteasome-mediated degradation,^11^ angiogenesis,^12^ calcium homeostasis,^13^ lipid metabolism^14^ and virus defense.^15^

Following brain injury, such as stroke, multiple cellular and physiological responses, which have the potential of influencing the extent of tissue damage, are activated. Within a few hours after an insult, a robust inflammatory response is evoked that involves production of cytokines, recruitment of innate immune cells to the site of damage and activation of resident brain macrophages called microglia.^16^ Concomitant with activation of innate immune responses, neurogenesis is also stimulated following brain injury, leading to increased proliferation of neural precursor cells in the subventricular and subgranular zones (SVZ and SGZ, respectively) and enhanced recruitment of migrating neuroblasts to sites of damaged brain tissue.^17^

During embryonic development of the brain cortex in mouse, PML regulates the transition from radial glia cells to basal progenitors.^18^ In addition, PML is required for maintenance and asymmetric commitment of hematopoietic stem cells.^19^ A recent study demonstrated a neuroprotective effect of PML in a mouse model of spinocerebellar ataxia 1.^11^ It is not clear, however, if PML participate in differentiation and/or tissue morphogenesis after injury in neonatal or adult animals.

In the present study we investigated the function of PML in protection and regeneration of brain tissue after damage. To achieve this, we studied neonatal wild-type and PML knockout mice subjected to HI. We discovered an essential role of PML in innate immune responses, inflammation and microglia activation during early stages after damage. In addition, we identified defects in HI-induced neuroblast production and reduced ratios of asymmetrically committed pairs of transit-amplifying progenitors during brain tissue recovery. Consequently, PML deficiency led to increased tissue loss during the acute phase after HI and impaired restoration of striatum during recovery.

## Results

### PML protects subcortical brain structures against HI-induced damage

To investigate a potential role of PML in protection and regeneration of tissue after injury, we induced cerebral ischemia by permanent occlusion of the left common carotid artery followed by systemic hypoxia in wild-type and PML-depleted mice at postnatal day 9 (P9). In this model, brain damage is produced in the hemisphere ipsilaterally to the occluded artery while the contralateral side of the brain is left undamaged.^20, 21, 22^

**Figure 1:**
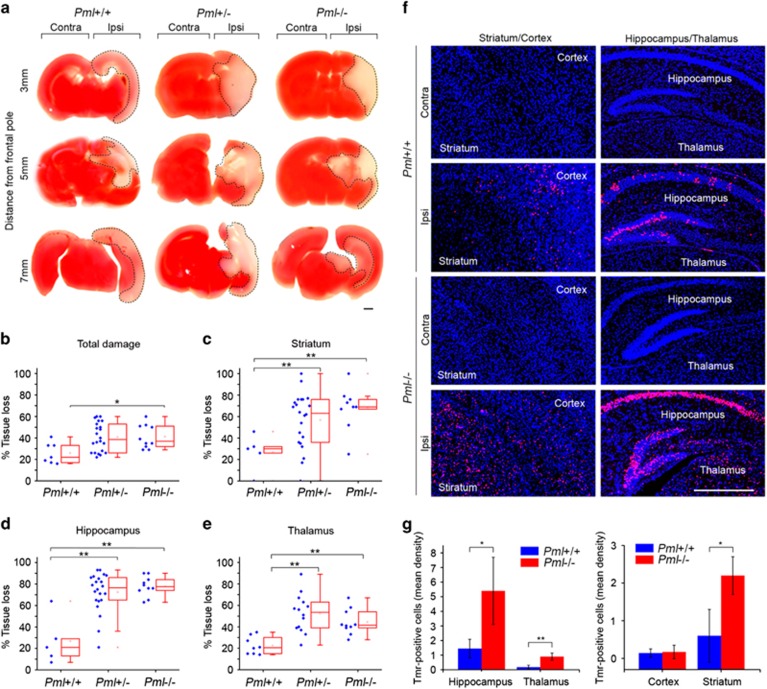
PML protects the brain from HI-induced tissue loss and apoptosis. (**a**) Representative TTC staining of brain sections 24 h after HI. Infarct area is indicated by dotted line. Scale bar 1 mm. (**b**–**e**) Quantification of infarct size in total brain and indicated brain subcompartments. For each mouse quantification was performed on six coronal sections. Mean value±standard deviation (S.D.) is shown, *n*=7–30 mice; one-way ANOVA with *post hoc* correction *P* (total)=0.012; *P* (hippocampus)<0.0001; *P* (thalamus)<0.0001; *P* (striatum)=0.019; asterisks indicate *t*-test for comparing indicated groups *P<0.05, **P<0.01. (**f**) Representative TUNEL staining of brain sections 24 h after HI. Left and right panels represent coronal sections on bregma 0.4 mm (showing striatum and cortex) and 1.6 mm (showing hippocampus and thalamus), respectively. Scale bar 1 mm. (**g**) Quantification of TUNEL-positive cells in indicated brain structures. Bars represent mean±S.D., *n*=4 mice, *P<0.05, **P<0.01

We first analyzed the extent of brain damage 24 h after HI by using 2,3,5-triphenyltetrazolium chloride (TTC) staining of brain sections (Figure 1a). Owing to an inherent variability of brain damage size after HI, these initial experiments were performed by first producing a random number of *Pml*+/+, *Pml*+/− and *Pml*−/− offspring through heterozygous (*Pml*+/−) mating, and by assessing the HI-induced damage blinded prior to genotyping. Despite relative large variations in the number of mice among different genotypes, these experiments revealed a significant increase in total brain tissue damage in *Pml*−/− compared with *Pml*+/+ mice (Figures 1a and b). Further analysis of specific brain subcompartments showed a significant increase in HI-induced tissue damage in striatum, hippocampus and thalamus in *Pml*−/− and *Pml* −/+ compared with *Pml*+/+ animals (Figures 1c–e).

We also investigated the extent of apoptotic cell death 1 day after HI using the TUNEL assay. In agreement with the data obtained by TTC analysis, we detected more apoptosis in the striatum, hippocampus and thalamus in *Pml*−/− compared with *Pml*+/+ mice. However, we did not detect significant differences in apoptosis between *Pml*+/+ and *Pml*−/− mice in the cortex (Figures 1f and g). Together, these data show that PML promotes neuroprotection after HI-induced damage and that this function of PML is particularly important for reducing the extent of injury within subcortical regions of the brain such as hippocampus, thalamus and striatum.

### PML regulates HI-induced innate immune responses

To identify the molecular mechanism involved in PML-mediated neuroprotection after HI-induced damage, we performed genome-wide expression analysis of sham-treated and HI-treated *Pml*+/+ and *Pml*−/− animals using RNA sequencing (RNA-Seq) 4 h after HI. We focused on the hippocampus since this subcompartment showed the most pronounced differences in HI-induced damage between wild-type and PML-deficient mice (Figure 1d).

**Figure 2:**
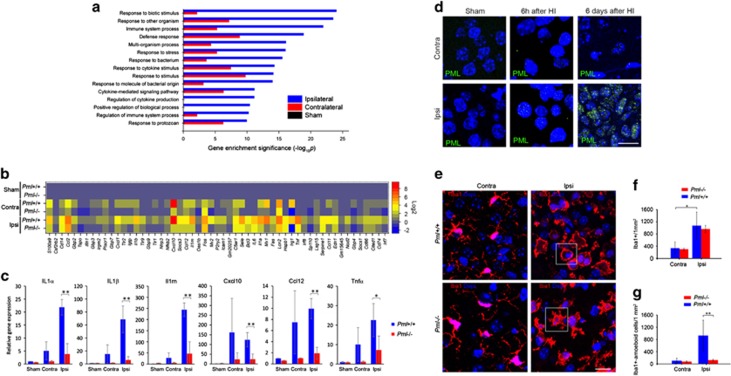
PML regulates HI-induced innate immune responses and microglia activation. (**a**) Differentially affected GO processes in PML wild-type *versus* PML-depleted brains. The 15 most significantly enriched GO processes are shown. Data were generated by genome-wide RNA-Seq analysis of total RNA samples isolated from hippocampus 4 h after HI. (**b**) Level plot showing fold induction of genes involved in response to biotic stimulus (the GO process with the highest enrichment significance). Log2 values for contralateral and ipsilateral brain hemispheres were normalized against Log2 values from sham-treated animals. (**c**) Real-time PCR analysis of selected inflammatory cytokines in sham-treated animals and HI-treated contralateral and ipsilateral hemispheres 4 h following HI. Bars represent mean relative expression±S.D., *n*=4 mice, *P<0.05, **P<0.01. (**d**) Confocal images showing the striatum of HI treated and the corresponding area in the striatum of sham (HI-untreated) brain stained with anti-PML antibody. DAPI staining is shown in blue. Images represent projections of multiple confocal z-sections. Scale bar 10 *μ*m. (**e**) Confocal images showing microglia detected by Iba1-specific antibodies (red) in contralateral and ipsilateral hemispheres of mouse brains 24 h after HI. DAPI staining is shown in blue. Images represent projections of multiple z-sections. Representative cell morphologies of ramified and amoeboid microglia in ipsilateral hemispheres after HI are indicated by white rectangles. Scale bar 10 nm. (**f** and **g**) Quantification of total microglia (**f**) and microglia exhibiting amoeboid morphology (**g**). Corresponding fields of ipsilateral and contralateral striatum within four non-adjacent coronal sections were analyzed (bregma 0.2–0.4 mm); *P<0.05, **P<0.01; data represent mean±S.D., *n*=3–4 mice

We identified 103 genes that were differentially regulated between *Pml*−/− and *Pml*+/+ hippocampus subjected to sham treatment. In mouse brains subjected to HI, the number of differentially regulated genes identified by RNA-Seq was 307 on the contralateral and 393 on the ipsilateral hemisphere. Gene ontology (GO) enrichment analysis of differentially expressed genes in the ipsilateral hemisphere revealed an overrepresentation of GO terms related to innate immunity (Figure 2a and Table 1). ‘Response to biotic stimulus' was the most highly ranked of GO processes and the genes within this group are displayed in the level plot shown in Figure 2b.

To validate the results obtained by RNA-seq analysis, we analyzed a selection of cytokines, including IL1*α*, IL1*β*, IL1RN, CXCL10, CCL12 and TNF*α*, which are known to have key roles in innate immunity and inflammation, by real-time PCR. In agreement with the RNA-seq analysis, this experiment revealed reduced expression of cytokine-encoding genes in PML defective compared with wild-type mice 4 h after HI (Figure 2c). Notably, several of the genes involved in activation of cytokine production overlap with the GO grouped genes involved in negative regulation of apoptosis (Table 1). This observation may, in part, explain the increased apoptosis observed in subcortical regions of PML-depleted mice after HI.

### PML-dependent microglia activation after HI

To investigate PML protein expression in the progression of HI, we immunofluorescently labeled coronal brain sections of untreated (sham), 6 h and 6 days after HI using a PML-specific antibody. There is no PML detected in the nucleus of untreated animals. However, 6 h after HI, we detected a weak nuclear PML staining pattern consisting of numerous small foci. Comparison between ipsilateral and contralateral hemispheres at this early time point did not reveal detectable differences in PML intensity and distribution (Figure 2d and Supplementary Figure S1). Analysis of sections acquired 6 days after HI, however, revealed a dramatically different PML signal in the ipsilateral compared with the contralateral side of the brain. While the contralateral hemisphere appeared to retain a staining pattern similar to that seen after 6 h, the ipsilateral striatum exhibited increased PML signal intensity in the focus of ischemic damage (Supplementary Figures S1a and b). In addition, PML bodies appeared to be larger in size within these cells (Figure 2d).

We next analyzed expression of PML at the mRNA level in different brain regions before and after ischemia. In untreated animals, the highest levels of *Pml* gene expression were found in the striatum and hippocampus, while the thalamus and hypothalamus showed the lowest levels (Supplementary Figure S3a). Four hours after HI, the subcortical regions of the brain, including hippocampus and striatum exhibited slightly reduced PML mRNA levels in both brain hemispheres (Supplementary Figure S3b). This result shows that the increased PML signal detected by IF 6 h after HI does not result due to increased PML gene expression. The stronger PML signal in these samples could be due to post-transcriptional regulation. Alternatively, the increased staining intensity could be due to increased recruitment of soluble PML to PML bodies.

Microglia are specialized brain macrophages that respond to infections or injury in a spatiotemporal manner.^23^ When challenged by HI, these cells secrete cytokines, proliferate and migrate towards the damaged brain tissue where they have the potential of exerting both neurotoxic and neuroprotective functions.^23, 24, 25, 26^ This is accompanied by characteristic changes in microglia morphology, which involve increased cell size, loss of ramifications and gain of amoeboid morphology.^27^ To investigate a role of PML in microglia-mediated inflammatory responses we immunofluorescently labeled brain sections from *Pml*−/− and *Pml*+/+ mice using antibodies against the microglia-associated protein Iba1. PML depletion did not significantly affect microglia proliferation following HI, as the total number of these cells was approximately the same in *Pml*+/+ and *Pml*−/− brains 24 h (Figures 2e and f) and 3 days (Supplementary Figures S4a and b) following injury. In addition, both wild-type and PML-depleted brains contained microglia with normal morphology (ramified) in the contralateral, non-damaged brain hemisphere after HI (Figures 2e and f and Supplementary Figures S4a and b). However, we observed striking differences in microglia morphology in the ipsilateral hemisphere 24 h (Figures 2e and g) and 3 days (Supplementary Figures S4a and b) after HI. Whereas wild-type microglia became larger, lost ramifications and developed amoeboid morphology, *Pml*−/− microglia partially retained the ramified morphology characteristic of resting microglia (Figures 2e and g and Supplementary Figures S4a and b). Further, microglia in the damaged *Pml*+/+ brain expressed CD68, a marker of phagocytic microglial activation 3 days after HI, while *Pml*−/− microglia remained CD68 negative (Supplementary Figures S4c and d).

Thereafter, we identified and analyzed microglia in the contralateral and ipsilateral hemisphere at the different stages of activation using defined criteria for morphology and orientation according to the previously described ‘spider-effect' model.^28^ We applied gated stimulated emission depletion (gSTED) microscopy to precisely address changes in size, morphology and numbers of PML bodies at different stages of microglia activation. While most microglia in the resting 1A stage had no detectable PML, the size of these nuclear bodies increased in parallel with a more advanced microglia activation phenotype (Supplementary Figures S2a and b). These data show that the size of PML bodies correlate directly with microglia activation.

### PML promotes brain tissue regeneration after HI

**Figure 3:**
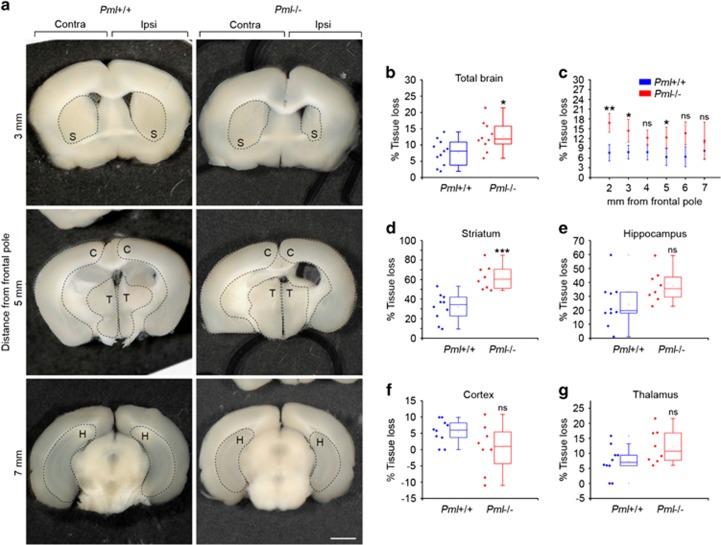
PML promotes brain tissue regeneration in the striatum after HI. (**a**) Coronal brain sections from bregma levels 0.4, 1.4 and 3.5 mm (3, 5 and 7 mm from the frontal pole, respectively) 42 days following HI. (S) striatum, (C) cortex, (T) thalamus and (H) hippocampus are outlined. Scale bar 1 mm. (**b**) Quantification of total brain tissue loss 42 days after HI; *n*=10–11 mice, data show mean±S.D., *P=0.013. (**c**) Tissue loss within coronal brain sections 2–7 mm from the frontal pole 42 days after HI. Bars represent mean±S.D., *n*=10–11 mice. *P<0.05, **P<0.01. (**d–g**) Quantification of tissue loss within indicated brain structures 42 days following HI. Bars represent mean±S.D., *n*=10–11 mice, ***P=0.0001; NS, not significant

To investigate long-term effects of PML depletion after brain injury, we measured brain tissue volume in contralateral and ipsilateral hemispheres 42 days following HI. At this time point the tissue loss was partially restored in both genotypes. However, we detected significantly more total brain tissue deficit in PML-negative compared with PML-positive mice (Figures 3a and b). Notably, the most profound differences between the two genotypes were observed in the rostral portions of the forebrain, 2 mm from the frontal pole (Figure 3c). Analysis of specific anatomical brain structures revealed that the majority of tissue loss in PML-depleted mice was confined to the striatum (Figures 3a and d). Analysis of hippocampus, thalamus and cortex revealed smaller nonsignificant differences between *Pml*+/+ and *Pml*−/− mice (Figures 3e–g). These data further demonstrate the importance of PML in protection against HI-induced brain damage and suggest that PML may be particularly important for protection and restoration of corpus striatum.

### PML promotes *de novo* production of neuronal progenitors

**Figure 4:**
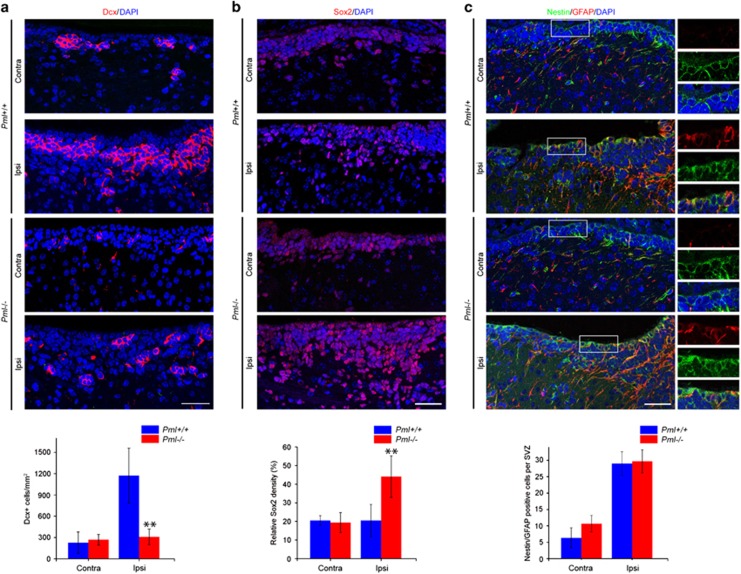
PML promotes *de novo* production of neuronal progenitors. (**a**) Confocal images showing Dcx-positive cells in ipsilateral and contralateral SVZ of *Pml*+/+ and *Pml*−/− mouse brains 6 days following HI. Images represent projections of multiple confocal z-sections. DAPI staining is shown in blue. The graph shows quantification of Dcx-positive cells 0–50 *μ*m from the ependym. The bars represent mean±S.D., *n*=4 mice, **P=0.007. Scale bar 30 *μ*m. (**b**) Confocal images showing Sox2-positive cells in ipsilateral and contralateral SVZ of *Pml*+/+ and *Pml*−/− mice. The graph shows integrated density of Sox2-positive cells relative to DAPI-stained cells. Cells 0–200 *μ*m from the ependym were analyzed. The bars represent mean±S.D., *n*=4 mice, **P=0.0012. Scale bar 30 *μ*m. (**c**) Confocal images showing IF-stained ipsilateral and contralateral SVZ 6 days after HI. Antibodies specific for Nestin and GFAP were used. DAPI is shown in blue. The graph represents total numbers of cells co-expressing Nestin and GFAP in the SVZ. Cells 0–200 *μ*m from the ependym were analyzed. The bars show mean±S.D., *n*=4 mice. Scale bar 30 *μ*m

The specific effect of PML depletion on striatal tissue volume that we observed 42 days after HI (Figure 3) suggested that PML may play a role in HI-induced neurogenesis. In agreement with this, PML have previously been shown to regulate differentiation of neural precursor cells during embryonic brain development in mouse.^18^ To investigate this, we analyzed brain sections from wild-type and PML-depleted mice at an early stage of the regeneration phase after HI (6 days) using antibodies that react with neural stem and precursor cells at different stages of differentiation. We detected an increased number of cells expressing doublecortin (Dcx), a protein expressed by migratory neuroblasts and immature neurons, in the SVZ of wild-type mice, but not in *Pml−/−* mice, after HI (Figure 4a). Conversely, quiescent neural stem cells and transit-amplifying progenitor cells, which are defined by Sox2 expression, were found to accumulate in the SVZ and striatum of PML-depleted mice but not in wild-type mice after HI (Figure 4b). Finally, cells that co-express GFAP and Nestin, markers for quiescent neural stem cells, were equally increased in the SVZ of *Pml* +/+ and *Pml* −/− mice after HI (Figure 4c). We also analyzed cell proliferation using the mitotic marker phospho-histone H3 (PHH3). This experiment revealed an increased number of mitotic cells in the ipsilateral *versus* contralateral hemisphere in both wild-type and PML-depleted brains 6 days after HI (Supplementary Figure S5a). Thus, loss of PML does not severely impede the overall proliferation capacity of cells in these regions of the brain. Altogether, these data showed that PML promotes *de novo* production of neuronal progenitors at the delayed stage after HI.

### PML promotes differentiation of Sox2-positive neural precursors into neuronal cell lineage

**Figure 5:**
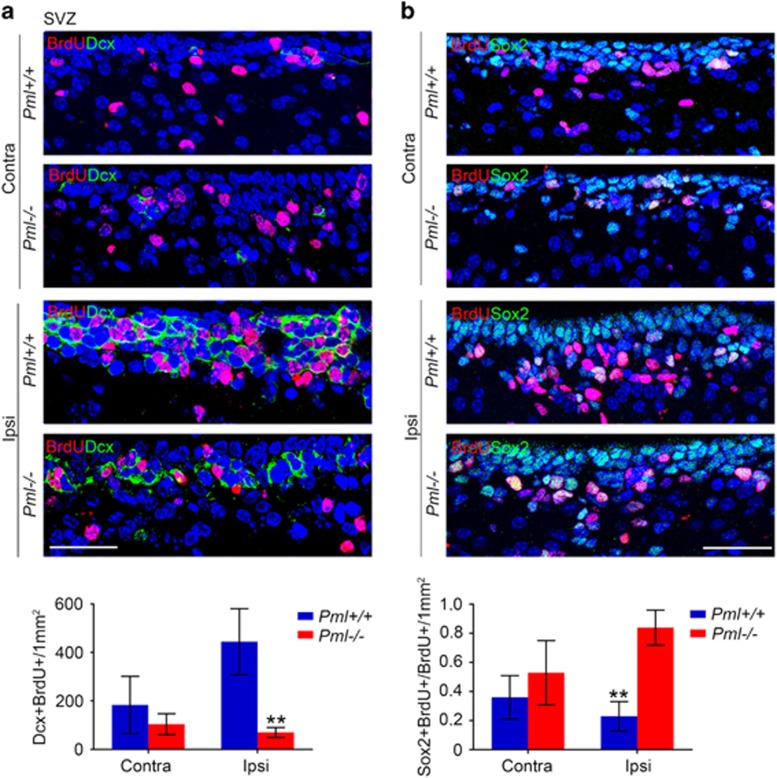
Differentiation of PML-deficient neural precursor cells accumulates Sox2-positive neural precursors. (**a**) IF showing BrdU- and Dcx-positive cells in ipsilateral and contralateral SVZ of *Pml*+/+ and *Pml*−/− mouse brains 6 days following HI. The BrdU injections were applied in the period between day 3 and day 7 after HI. Images represent projections of multiple confocal z-sections. DAPI staining is shown in blue. The graph shows quantification of cells double-positive for BrdU and Dcx 0–50 *μ*m from the ependym. The bars represent mean±S.D., *n*=4 mice, **P<0.01. Scale bar 30 *μ*m. (**b**) Confocal images showing BrdU- and Sox2-positive cells in ipsilateral and contralateral SVZ of *Pml*+/+ and *Pml*−/− mouse brain 6 days following HI. The graph shows quantification of BrdU- and Sox2 double-positive cells relative to the total number of BrdU-positive cells. Images represent projections of multiple confocal z-sections. DAPI staining is shown in blue. Cells 0–200 *μ*m from the ependym were analyzed. The bars represent mean±S.D., *n*=4 mice, **P<0.01. Scale bar 30 nm

To address if PML depletion causes arrest in differentiation of *de novo* produced neural precursors, we treated *Pml+/+* and *Pml−/−* mice with HI followed by intraperitoneal injections of the thymidine analog 5-bromo-2-deoxyuridine (BrdU), which is incorporated during the S-phase of the cell cycle. Four consecutive BrdU injections were performed at 24 h intervals from day 3 to 7 after HI. We analyzed SVZ cells for expression of BrdU, Dcx and Sox2. In accordance with the PHH3 staining (Supplementary Figure S5a), the total number of BrdU-positive cells increased equally in both genotypes (Supplementary Figure S5b). In the ipsilateral hemisphere of *Pml+/+* brains, most of the BrdU-positive cells were observed to express Dcx. The BrdU-positive cells detected in *Pml−/−* brains, on the other hand, were mostly devoid of this neuronal precursor marker protein (Figure 5a). Conversely, most of the neural precursors in the SVZ were Sox2 negative in *Pml+/+* and Sox2 positive in *Pml−/−* (Figure 5b).

We also analyzed *in vitro*-cultured neuronal stem cells (neurospheres) by IF using neural cell-specific markers. In agreement with a previous study,^18^ we found that PML-depleted neurospheres generally were larger, expressed higher levels of Nestin and lower levels of GFAP and Tuj1 compared with wild-type neurospheres (Supplementary Figure S6). Additionally, *Pml−/−* neurospheres are enriched with Sox2 positive, but depleted for Dcx-positive cells (Supplementary Figure S6).

Together these data confirm a role of PML in promoting differentiation of Sox2-positive neural precursors into neuronal progenitors during HI-stimulated neurogenesis.

### PML regulates asymmetric commitment of newly divided neural precursor daughter cells

**Figure 6:**
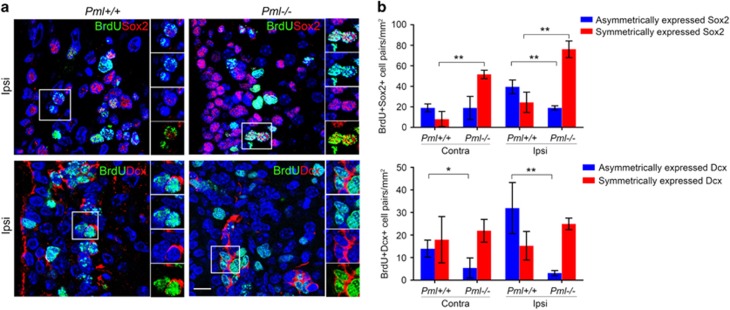
PML regulates asymmetric commitment of newly divided neural precursor daughter cells. (**a**) IF showing BrdU-, Sox2- and Dcx-positive cells within the SVZ 6 days following HI. Images represent projections of multiple confocal z-sections. The rectangles highlight pairs of BrdU-positive daughter cells presented on close up images. Scale bar 10 nm. (**b**) Quantification of symmetric and asymmetric divisions of BrdU-positive cells. For each sample, corresponding fields of ipsilateral and contralateral SVZ within three non-adjacent coronal sections (0.2–0.7 mm) were analyzed. Pairs of BrdU-positive cells containing at least one Dcx – or at least one Sox2-positive cell – were included in the analysis. The bars represent mean±S.D., *n*=4 mice, *P<0.05, **P<0.01

The Sox2-positive neural precursor cells have previously been shown to contribute to Dcx-positive neuroblasts through asymmetric cell division.^29^ In addition, PML has recently been shown to regulate asymmetric division of mouse hematopoietic stem cells. To investigate a role of PML in regulation of asymmetric commitment of neural precursors, we studied daughter cell pairs derived from BrdU-labeled neural precursors.^30^ We considered pairs of BrdU-positive cells as sister cells if they: (i) showed mirror morphologies (which characterizes daughter cells shortly after cell division), (ii) showed similar configuration and intensity of BrdU labeling, (iii) displayed similar configuration of chromatin as determined by DAPI counterstaining, and (iv) were their closest BrdU-positive partners. We used a BrdU-specific antibody in combination with anti-Dcx or anti-Sox2 antibodies to identify BrdU-positive cell pairs and to distinguish between symmetric *versus* asymmetric cell divisions in the SVZ (Figures 6a and b). This experiment revealed significantly lower ratios of asymmetric Sox2 and Dcx expression in PML-depleted *versus* wild-type mice (Figures 6a and b). The data demonstrate that PML promotes asymmetric division of transit-amplifying progenitors.

## Discussion

The physiological response to brain injury consists of an acute phase, which involves activation of innate immune responses, and a delayed phase, which involves neurogenesis, tissue repair and morphogenesis. To better understand the processes involved in protection and repair of tissue after brain injury it is important to identify the proteins involved and to understand their mechanism of action. In the present study we have shown that PML has an important role in minimizing the consequences of brain injuries. In agreement with this, we identified PML as an important mediator of innate immunity, inflammation and neurogenesis, physiological responses that are activated at different stages after an insult and that have the potential to influence brain injury outcome.

The increase in apoptosis and tissue loss observed after HI in PML-depleted mice was unexpected in view of previous studies demonstrating proapoptotic functions of PML.^6, 13, 31, 32^ The higher levels of cell death observed in the striatum, thalamus and hippocampus in PML-depleted compared with wild-type mice following HI may be explained by the observed requirement of this protein for HI-induced cytokine production. Thus, the function of PML in regulation of innate immunity and inflammatory responses may override other functions of PML in proapoptotic pathways. In support of this, the GO analysis of the RNA-seq data obtained from PML-depleted and wild-type hippocampus after HI revealed significant enrichment of differentially expressed genes in GO processes such as negative regulation of apoptosis and negative regulation of programmed cell death.

Previous studies have indicated a role of PML in promoting inflammation through regulation of cytokines such as IL1*β*, IFN*γ*, INF*β* and IL6.^33, 34, 35, 36, 37, 38^ The present study suggests a more fundamental role of PML in stimulating a broad spectrum of key cytokines involved in innate immunity and inflammatory pathways. PML may in part control HI-induced cytokine production through microglia activation. In agreement with this, PML-depleted mice failed to produce microglia with the characteristic activated morphology and detectable levels of CD68 at the site of tissue damage. In addition, we observed increased size of PML bodies in these cells that correlated with defined stages of HI-induced microglia activation.

The mature neuronal cells that are derived from neural progenitors may participate in repopulation and rebuilding the injured brain tissue.^39, 40^ In the present study we discovered a specific defect in the ability of PML-depleted mice to restore neural deficits in the striatum after HI. Noteworthy, a behavioral study would be needed to address if impaired tissue restoration in PML-depleted mice affect neurological recovery. However, a reliable behavioral assay for mice at such a young age is not available at present. These mice were also found to be defective in *de novo* generation of migratory Dcx-positive neuronal progenitors and exhibited increased accumulation of Sox2-positive transit-amplifying progenitors in the SVZ and striatum. Thus, the role of PML in striatum regeneration after HI may be directly linked to a function of this protein in commitment of Sox2-positive neural precursors into Dcx-positive neuronal progenitors. Finally, microglia could contribute to cerebral-ischemia-induced neurogenesis. Activating microglia is proposed to stimulate *ex vivo* neural stem cell proliferation and differentiation into oligodendrocytes and neurons. Additionally, microglia that stimulate neurogenesis by production of IGF-1 accumulate in the SVZ of a stroke-injured brain.^41^ Nevertheless, in the present study, arrest in differentiation of PML-depleted transit-amplifying precursors is confirmed in neurosphere assays *in vivo*, regardless of microglia presence. Thus, the present study suggests an inflammation-independent effect of PML on production of neuronal progenitors.

The observed defect of *Pml−/−* mice in production of neuronal progenitors after tissue damage may be related to previous work showing that PML controls brain cortex development during embryogenesis.^18^ In this study PML was found to stimulate the transition between radial glial cells and intermediate progenitors. The present work suggests that the defect in neuronal progenitor development in PML-depleted mice may stem from a role of this protein in regulating the balance between asymmetric and symmetric commitment of dividing neural precursors. In agreement with this, PML has been shown to contribute to renewal of hematopoietic stem cells through stimulation of differential commitment of newly divided daughter cells. In the neocortex of developing mice embryos, radial glia cells have been shown to divide asymmetrically to simultaneously stimulate formation of a progeny stem cell and a neural precursor.^16, 42^ Importantly, upon stimulation of neurogenesis dividing Sox2-positive cells are known to self-renew and give rise to Dcx-positive neuronal progenitors through asymmetric commitment of daughter cells.^29^ Thus, PML might have a direct function in injury-induced neurogenesis by inducing asymmetric segregation of transit-amplifying progenitors.

The present paper describes key functions of PML in protection and neurogenesis following HI-induced damage. While PML contributes to prevention of apoptosis and tissue loss at early stages after ischemia through activation of pathways involved in innate immunity and inflammation, the role of this protein during recovery may be linked to asymmetrical commitment of transit-amplifying progenitors.

## Materials and Methods

### Mice

129Sv *Pml*−/− mice^43^ were obtained from the National Cancer Institute Repository in Frederick, MD, USA. Mice are backcrossed to C57BL/6 mice for eight generations. Wild-type controls were C57BL6. For all experiments described in this study postnatal day 9 (P9) mice were used. The mice were bred and housed in a 12 h light/dark cycle at the Department of Comparative Medicine, Oslo University Hospital, Rikshospitalet, Norway, with a diet of pellets and water *ad libitum*. The Norwegian Animal Research Authority approved all experimental procedures used in the present study.

### Hypoxia and ischemia

Cerebral hypoxia and ischemia were induced by permanent occlusion of the left common carotid artery (CCA) prior to systemic hypoxia as previously described.^20^ In brief, P9 mice were anesthetized with isoflurane (4% induction in the chamber followed by exposure to 2.5% Isoflurane maintenance equilibrated with an ambient of air and oxygen in the ratio 2:1) followed by skin incision at the anterior midline of the neck. Following artery preparation, a needle was placed into the artery and monopolar cauterization (Hyfrecator 2000; ConMed, Utica, NY USA) was carried out at a power of 4.0 W to electrocoagulate the artery. Skin incisions were then closed by absorbable sutures (Safil 8-0, DRM6; B. Braun Melsungen Ag, Hessen, Germany). The entire operation, from skin incision to closure, lasted for approximately 5 min. Following a recovery period for 90 min the surgically treated mice were exposed to an hypoxic, humidified atmosphere containing 10% oxygen balance nitrogen (Yara, Oslo, Norway) for 60 min at 36.6 °C. The pups were returned to their dam and after 6 or 24 h, the brains were retrieved and prepared for immunohistochemistry, microarray or TTC staining. Sham-treated animals were subjected to anesthesia, skin incision with suturing, but not CCA occlusion and hypoxia.

### Quantification of tissue loss after HI

Mice treated for 24 h and 42 days by HI were killd and forebrains were removed from the scull and freed from dura mater and vascular tissue. The brains were transferred to a precooled brain mold immersed in ice-cold PBS. Two percent TTC (T8877, Sigma) were prepared and kept isolated from light. Coronal sections with an even thickness of 1 mm per slice were prepared on ice, using an adult brain slicer (51-4984; Zivic Instruments, Pitsburgh, PA, USA). Sections were soaked in 2% TTC in PBS for 30 min on room temperature and subsequently fixed in 4% PFA (Sigma-Aldrich, St. Louis, MO, USA; 15,812-7) in PBS at 4 °C for a maximum of 3 days. Following fixation photos were made using a digital camera (Nikon D80) on approximately equal flash-mediated lightning and pictures were analyzed using image J software (NIH, San Francisco, CA, USA). Quantification of tissue loss after HI was carried out as previously described.^20^ Briefly, the infarct area was calculated by subtracting the area of undamaged, TTC positive tissue in the ipsilateral hemisphere from that of the intact contralateral hemisphere, thereby correcting for brain edema. Relative size of the damage was expressed as percent of the contralateral hemisphere. Total volume loss as well as tissue loss within specific brain structures was calculated by modified Cavalieri's principle, using the formula *V*=∑*APt* where *V* is the total volume, ∑*A* is the sum of the areas measured, *P* is the inverse of the section sampling fraction and *t* is the section thickness. For brains analyzed 42 days after HI, coronal sections were prepared as aforementioned above, but without TTC staining. Sections were fixed in 4% PFA in PBS for 30 min and 10% formalin for 24 h and photos were taken. Tissue loss was calculated by subtracting the total volume, section volume or structure volume of the ipsilateral hemisphere from that of the contralateral hemisphere.

### Immunohistochemistry

Mice were anesthetized and transcardially perfused prior to brain removal and fixation in 4% paraformaldehyde for 24 h.^20^ Following fixation in 10% formalin and embedding in paraffin, 4-*μ*m-thick coronal sections through the entire forebrain were made using a microtome (ThermoScientific, Waltham, MA, USA). Samples were dehydrated in Neoclear (Millipore, Darmstad, Germany) followed by rehydration in an EtOH gradient (100%, 3 min; 100%, 3 min; 96%, 1 min and 70% 1 min) and subsequently incubated at 100 °C in citrate antigen-retrieval buffer (pH 6.0), containing 0.05% Tween 20 (Sigma) for 3 min under increased pressure, followed by nuclear permeabilization with 0.25% Triton X-100 in PBS for 45 min. Sections were subsequently incubated in the presence of blocking buffer containing 5% goat serum, 5% bovine serum albumin and 0.1% Tween 20 in PBS for 30 min followed by incubation with primary antibodies diluted in blocking buffer at 4 °C over night. After three washes in PBS containing 0.1% Tween 20, staining with secondary antibodies was performed for 90 min at 37 °C. After three washes in PBS (5 min each) samples were washed in Vectashield mounting medium containing DAPI (Vector Laboratories, Burlingame, CA, USA). Microscopy was carried out using a Leica SP8 confocal microscope equipped with × 40 oil immersion lens. gSTED images were acquired using a 3D Leica TCS SP8 gated STED microscope and × 100 oil immersion lens.

### BrdU incorporation

For the BrdU incorporation mice were treated with hypoxic ischemia as described above and 0.1 mg/g of BrdU was injected into the peritoneum on days 3–7 after ischemia, at 24- h intervals. Animals were transcardially perfused 2 h after the last injection and brains were fixed and stained as described above.

### Antibodies

The primary antibodies used were mouse anti-GFAP, 1 : 500 (G3893; Sigma); rabbit anti-Dcx, 1 : 2000 (ab18723; Abcam, Cambridge, UK); rabbit anti-phospho-histone H3, 1 : 800 (06–570; Millipore); rabbit anti-Iba1, 1 : 2000 (019–19741; Wako, Richmond, USA); mouse anti-Nestin, 1 : 200 (MAB353; Millipore); rabbit anti-Neuron-specific beta-III Tubulin (Tuj1) antibody, 1 : 200 (MAB1195; R&D Systems, Abingdon, UK); mouse anti-NeuN, 1 : 400 (MAB377; Millipore); rabbit anti-Sox2, 1:200 (ab97959, Abcam); rat anti-BrdU, 1:200 (Abcam); mouse anti-PML, 1 : 200 (36.1-107; Millipore); rabbit anti-GFAP 1 : 400 (Abcam). The secondary antibodies used were Alexa Fluor 488 (Invitrogen) and Alexa Fluor 555 (Invitrogen, Waltham, MA, USA), both at dilutions of 1 : 400.

### TUNEL staining

TUNEL staining was performed using the In Situ Cell Death Detection Kit (TMR-Red) from Roche (Basel, Switzerland). Briefly, 4-m-thick, non-adjacent coronal brain sections were warmed in an incubator at 57 °C for 10 min, dehydrated in Neoclear and subsequently rehydrated in an EtOH gradient (100%, 1 min; 100%, 1 min; 96%, 1 min and 70% 1 min). After a 3 min wash in lukewarm running tap water, samples were incubated in the presence of 0.1% Proteinase K in 10 mM Tris-HCl, pH=7.5, 37 °C for 20 min. Samples were washed in PBS two times for 3 min at room temperature and subsequently incubated for 60 min at 37 °C in the presence of 20 *μ*l TUNEL reaction mix containing enzyme and TMR (Tamra) red labeling solution at a 1 : 9 ratio. Samples were then washed three times 3 min in PBS at room temperature and subsequently mounted in Vectashield containing DAPI. Fluorescence images were generated using a fluorescent Zeiss Axio Observer.Z1 microscope (Cambridge, UK) with a × 5 air objective. Mean density of TUNEL-positive cells was quantified using the Image J software on two non-adjacent sections per bregma.

### Culturing and immunolabeling of neurospheres

Neurospheres were derived from SVZ of *Pml*+/+ and *Pml*−/− mice forebrain as previously described.^20^ After passing through a 70 *μ*m filter, cells were suspended at a concentration of 20 000/ml in T75 flasks containing proliferation medium (Neurobasal-A (Gibco, Waltham, MA, USA) supplemented with 2 mM l-glutamine (GlutaMAX; Gibco), 2% B-27 supplement (Gibco), 15 ng/ml recombinant human FGF basic (R&D Systems; 234-FSE) and 20 ng/ml EGF (R&D systems; 234-EG-200)). At day 7 of second passage (P2) 10 ml of even Neurosphere suspension was centrifuged at 1000 × *g* for 3 min at room temperature. Pellets were suspended in 45 *μ*l freshly thawed plasma (obtained from local blood bank) and 20 *μ*l Trombin (Sigma; T-4648). Coagulum with trapped neurospheres was fixed in 4% PFA in PBS for 24 h and transferred to Cellsafe+ Biopsy capsule (Chemi-Teknik AS, Oslo, Norway) prior to immersion in 10% formalin. Neurospheres were dehydrated and embedded into paraffin. Four- *μ*m-thick sections were prepared using a microtome (ThermoScientific) and subjected to deparafinization, antigen-retrieval and immunofluorescent staining as described under the Immunohistochemistry section.

### RTQ-PCR analysis

Total RNA was isolated from mouse hippocampus using the RNeasy Kit (Qiagen, Hilden, Germany) and cDNA was prepared from 1.0 *μ*g total RNA using the High Capacity cDNA reverse transcription kit (Applied Biosystems). Briefly, tissue was homogenized in lysis buffer RLT using Fastprep^R^-24 instrument, centrifuged for 3 min, 30 000 × *g* and supernatant was used for total RNA isolation. RNA concentration was measured by a NanoDrop Spectrophotometer. Quantitative real-time PCR was performed using the Power SYBR green PCR Master Mix (Applied Biosystems) in a 7900HT Fast Real-Time PCR System (Applied Biosystems): 10 min at 95 °C; 15 s at 95 °C; 1 min at 60 °C; repeat for 40 cycles. The ΔΔCT method was used to quantify the relative gene expression using *Gapdh* and *β*-actin as the internal reference. Primers were designed using Primer3 software and quality was determined with melting curves. Primer target sequences used are shown in the table (-F, forward; -R, reverse).


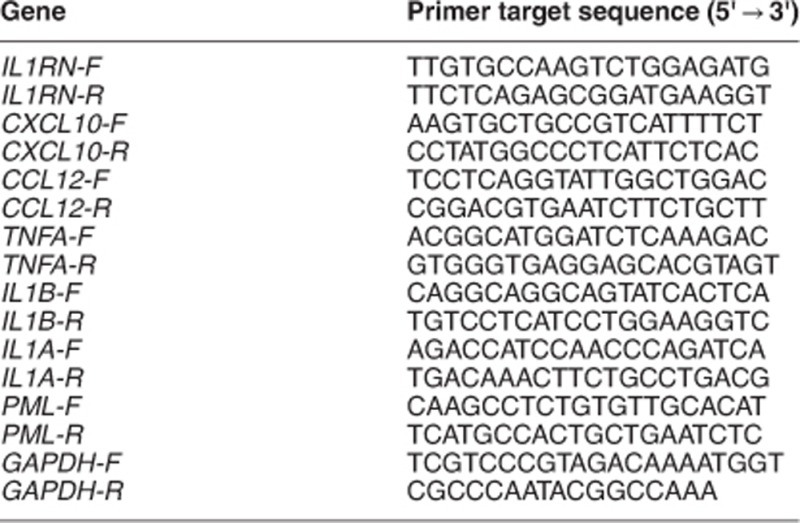


### RNA-seq analysis

RNA was isolated from hippocampus as aforementioned above under RTQ-PCR analysis. Pooled samples from four different animals per genotype (all females) were used as a source for library construction. Library construction, sequencing and data processing were performed through a commercially available service by Beijing Genomics Institute (BGI, Guangdong, China), China. Gene ontology enrichment analysis was performed on the filtered data using a hypergeometric distribution model. Level plot was designed by using RStudio (New Jersey, USA).

### Quantifications and statistical analysis

All quantifications were performed blinded, manually and using Image J software (NIH). Data are expressed as mean±S.D. and based on at least three independent experiments. One-way ANOVA with *post hoc* Geisser-Greenhouse correction was used when more than two groups are compared. Two-tailed *t*-test is used when two groups are compared. Correlation was analyzed by Pearson's coefficient. Differences were considered significant when *P* was less than 0.05.

## Figures and Tables

**Table 1:** Gene ontology biology process analysis of RNA-seq data GO processes differentially affected in wild-type and PML-depleted hippocampus after HI

**Gene ontology group**	**Sham-treated mice, PML**−/− ***versus* PML+/+ (*****P***-**values)**	**HI-treated mice, PML**−/− ***versus* PML+/+ (*****P***-**values)**	
		**Contralateral hemisphere**	**Ipsilateral hemisphere**
Response to cytokine stimulus	NS	3.36 × 10^−8^	9.1 × 10^−25^
Cytokine-mediated signaling pathway	NS	4.73 × 10^−7^	6.53 × 10^−12^
Positive regulation of cytokine production	NS	NS	8.51 × 10^−9^
Interferon-*β* response	NS	9.65x10^−9^	2.01 × 10^−7^
Response to wounding	NS	NS	5.6 × 10^−6^
Regulation of leukocyte migration	NS	NS	3.63 × 10^−6^
Inflammatory response	NS	NS	7.7 × 10^−5^
Response to interleuin-1	NS	NS	3.42 × 10^−5^
Negative regulation of apoptosis	NS	NS	0.00013
Negative regulation of cell death	NS	NS	0.00014

Abbreviation: NS, not significant. GO terms are ranked from top to bottom based on enrichment significance of wild-type *versus* PML-depleted ipsilateral hippocampus

## Abbreviations

PML or PML protein: promyelocytic leukemia protein
TUNEL: terminal deoxynucleotidyl transferase dUTP nick end labeling
IL1*α*: interleukin 1alpha
IL1*β*: interleukin 1beta
IL1RN: interleukin 1 receptor antagonist
CXCL10: C-X-C motif chemokine 10
CCL12: chemokine (C-C motif) ligand 12
TNF*α*: tumor necrosis factor alpha
APL: acute promyelocytic leukemia
gSTED: gated stimulated emission depletion microscopy
RARA: retinoic acid receptor alpha
TTC: triphenyltetrazolium chloride
Dcx: doublecortin
PHH3: phospho-histone H3
BrdU: bromodeoxyuridine
GFAP: glial fibrillary acidic protein
Tuj1: class III beta-tubulin
Iba1: ionized calcium-binding adapter molecule 1
HI: hypoxia–ischemia
SVZ: subventricular zone
Sox2: SRY (sex determining region Y)-box 2
GO: gene ontology
