# Supplementary material for: PML regulates neuroprotective innate immunity and neuroblast commitment in a hypoxic–ischemic encephalopathy model
Source: Cell Death Dis. 2016 Jul 28;7(7):e2320–. doi: 10.1038/cddis.2016.223 (PMC4973360; doi:10.1038/cddis.2016.223)
Supplement: Supplementary Information [file cddis2016223x1.docx]

**PML regulates neuroprotective innate immunity and neuroblast commitment in a hypoxic-ischemic encephalopathy model**

Vuk Palibrk^1, 2^, Rajikala Suganthan^3^, Katja Scheffler^1, 2^, Wei Wang^1, 2^, Magnar Bjørås^1,2,3^* and Stig Ove Bøe*^1^

^1^Department of Medical Biochemistry, Oslo University Hospital and University of Oslo, Oslo, Norway

^2^Institute for cancer research and molecular medicine, Norwegian University of Science and Technology, Trondheim, Norway

^3^Department of Microbiology, Oslo University Hospital, University of Oslo, Oslo, Norway

*Correspondences: Stig Ove Bøe,

Department of Medical Biochemistry

Oslo University Hospital

Songsvannsveien 20

0027 Oslo, Norway

Tel: +47 92600859

Fax: +47 27070902

Email: [stig.ove.boe@rr-research.no](mailto:stig.ove.boe@rr-research.no)

Magnar Bjørås,

Institute for cancer research and molecular medicine

Norwegian University for Science and Technology

Trondheim, Norway

Tel: +47 72836369

Email: [magnar.bjoras@ntnu.no](mailto:magnar.bjoras@ntnu.no)

**Supplementary figures**

**Supplementary figure S1. Expression of PML protein in the focus of ischemic damage and corresponding ipsilateral striatum:**

Confocal images showing PML bodies in striatum of *Pml+/+* sham, 6h and 6 days after HI. Focus of the damage is indicated by activated microglia and astrocyte, stained with anti-Iba1 (a) and anti-GFAP (b) antibodies respectively. Images represent projections from multiple confocal brain sections. Scale bar 10µm.

**Supplementary figure S2. Reorganization of PML bodies in activating microglia**

(a) *Left panel:* Confocal images showing microglia labeled by DAPI, anti-PML antibody and anti-Iba1 antibody in the different stages of activation cascade according to the “spider-effect” model. *Middle panel:* Same images as left panel with DAPI and anti-PML antibody staining (without anti-Iba1 staining). *Right panel:* gSTED images of the largest PML bodies at the different stages of microglia activation as indicated in the left and middle panel. Scale bar 1 μm.

(b) Size of the largest PML body within a nucleus of *Pml+/+* microglia at the different stages of activation; n=5 mice, 4-5 microglia per stage of activation were analyzed; one-way ANOVA with post-hoc correction is p<0,0001; Pearson’s correlation between size of the PML body and stage of activation: p=0,018 (correlation coefficient (CC) = 0,88); asterisks indicate t-test p-value for comparing indicated stages: **p (2A vs 4A)=0,0031, **p (2A vs 6A)=0,0024, **p=0,001;

**Supplementary figure S3. PML mRNA levels in brain**

(a) Relative PML gene expression in different brain regions 4 hours after hypoxic ischemia. Asterisks indicate difference related to cortex. Data represent mean ± SD, n=4, *p˂0.05, **p˂0.01. Ctx-cortex, Hc-hippocampus, Str-striatum, Th-thalamus, Hth-hypothalamus.

(b) Relative PML gene expression in different brain regions of sham (HI-untreated) and HI treated animals in the contra- and ipsilateral hemisphere 4h after hypoxic ischemia. The graph represents the expression in HI-treated related to sham-treated. Mean ± SD, n=4, *p˂0.05, **p˂0.01. Ctx-cortex, Hc-hippocampus, Str-striatum, Th-thalamus.

**Supplementary figure S4. Microglia activation 3 days after HI**

(a) Representative confocal microscope images showing Iba1 labeling of microglia cells 3 days after HI. DAPI staining is shown in blue.

(b) Quantification of total microglia (lower graph) and microglia exhibiting amoeboid morphology (upper graph). Corresponding fields of ipsilateral and contralateral striatum within four non-adjacent coronal sections were analyzed (bregma 0.2-0.4 mm). Data represent mean ± SD, n = 4 mice. *p<0.05.

(c) Representative confocal microscope images showing Iba1 (red) and CD68 (green) of ipsilateral striatum 3 days after HI. DAPI staining is shown in blue.

(d) Quantification of cells double - positive for Iba1 and CD68. Corresponding fields of ipsilateral and contralateral striatum within four non-adjacent coronal sections were analyzed (bregma 0.2-0.4 mm). Data represent mean ± SD, n = 4 mice. *p<0.05.

**Supplementary** **figure S5. HI-induced mitosis in SVZ and striatum.**

(a) Quantification of phospho-histone H3-positive (PHH3+) cells in striatum and SVZ of *Pml*+/+ and *Pml*-/- brains 6 days after HI. Data represent mean ± SD, n = 4 mice.

(b) Quantification of BrdU positive (BrdU+) cells in striatum and SVZ of *Pml+/+* and *Pml-/-* brains 6 days after HI. Data represent mean ± SD, n=4 mice.

**Supplementary figure S6. IF analysis of wild type and PML-depleted neurospheres.**

Confocal images showing neurospheres derived from the SVZ of P9 mice. A single confocal z-section is shown. Immunolabeled proteins are indicated. DAPI is shown in blue.
