# Supplementary figures and images for: PML regulates neuroprotective innate immunity and neuroblast commitment in a hypoxic–ischemic encephalopathy model
Source: Cell Death Dis. 2016 Jul 28;7(7):e2320–. doi: 10.1038/cddis.2016.223 (PMC4973360; doi:10.1038/cddis.2016.223)

Supplementary figure S1

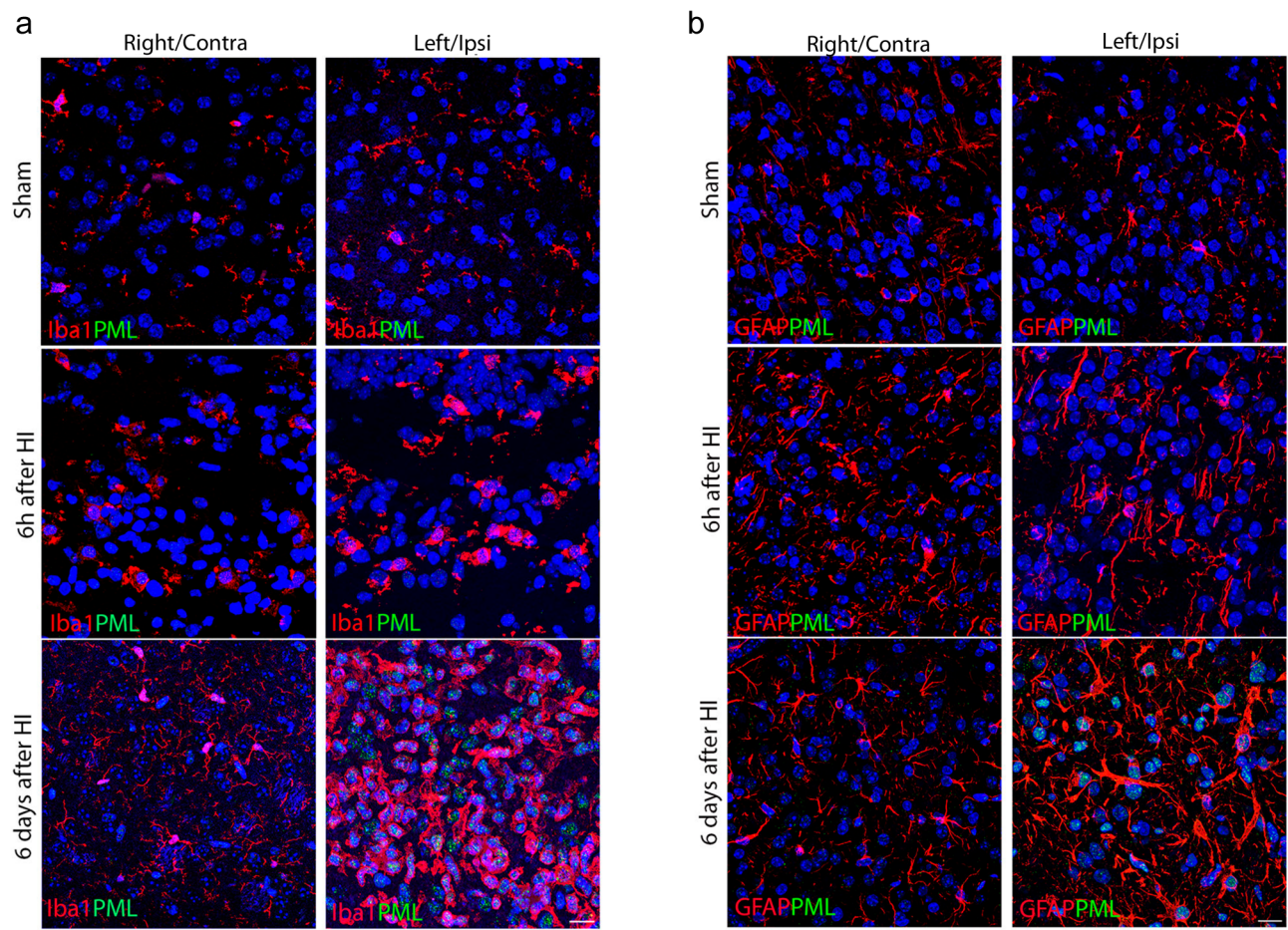

Supplement: Supplementary Figure S1 [file cddis2016223x2.pdf]

Supplementary figure S2

a

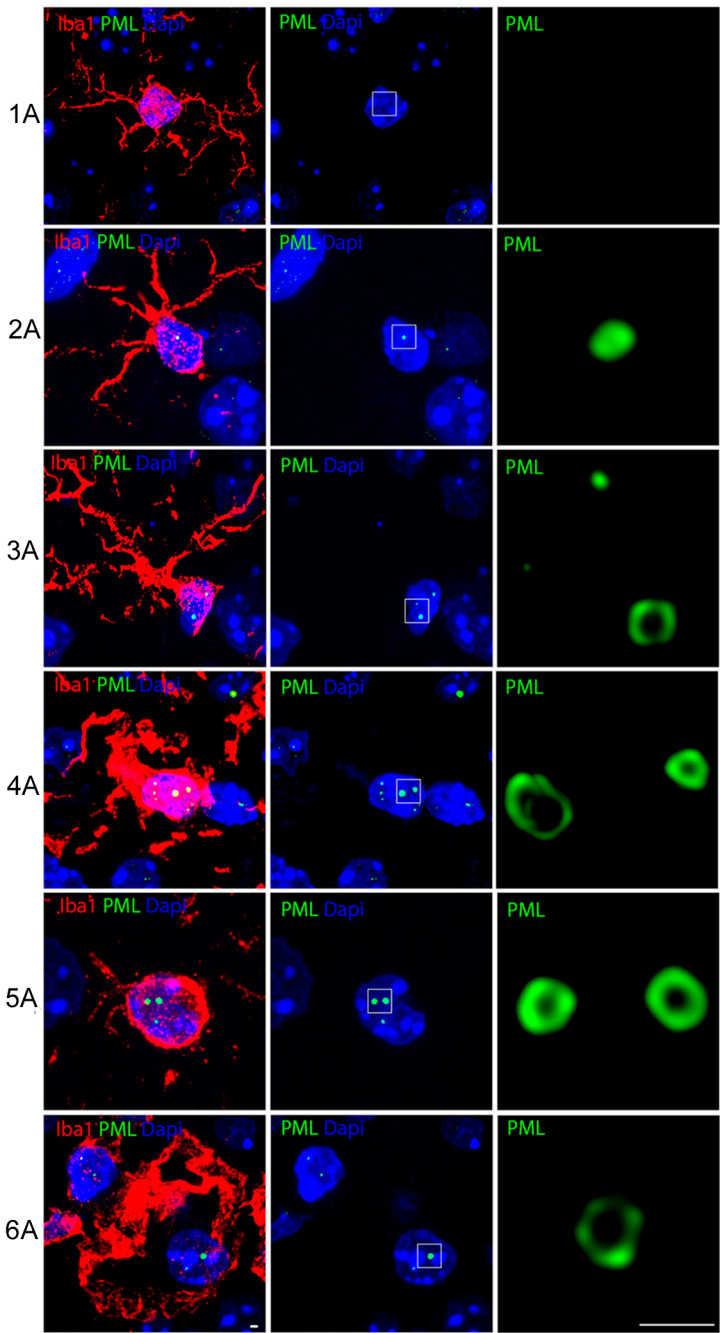

b

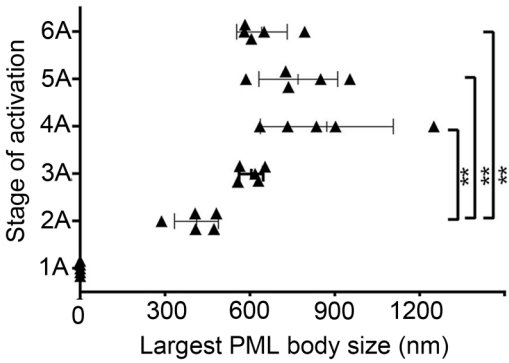

Supplement: Supplementary Figure S2 [file cddis2016223x3.pdf]

Supplementary figure S3

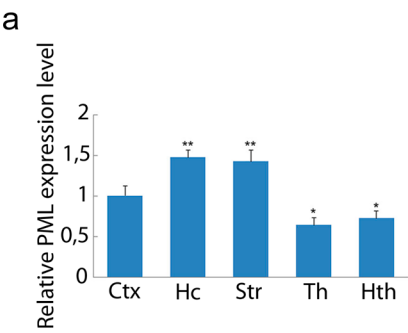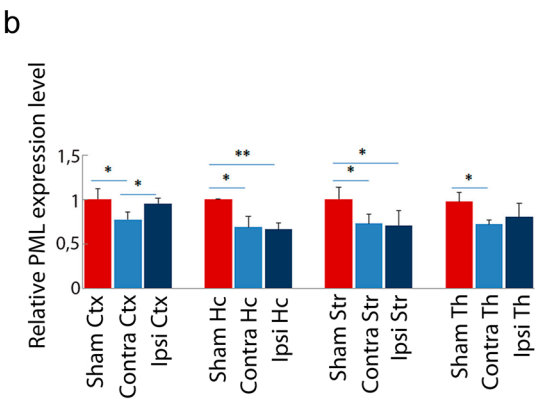

Supplement: Supplementary Figure S3 [file cddis2016223x4.pdf]

Supplementary figure S4

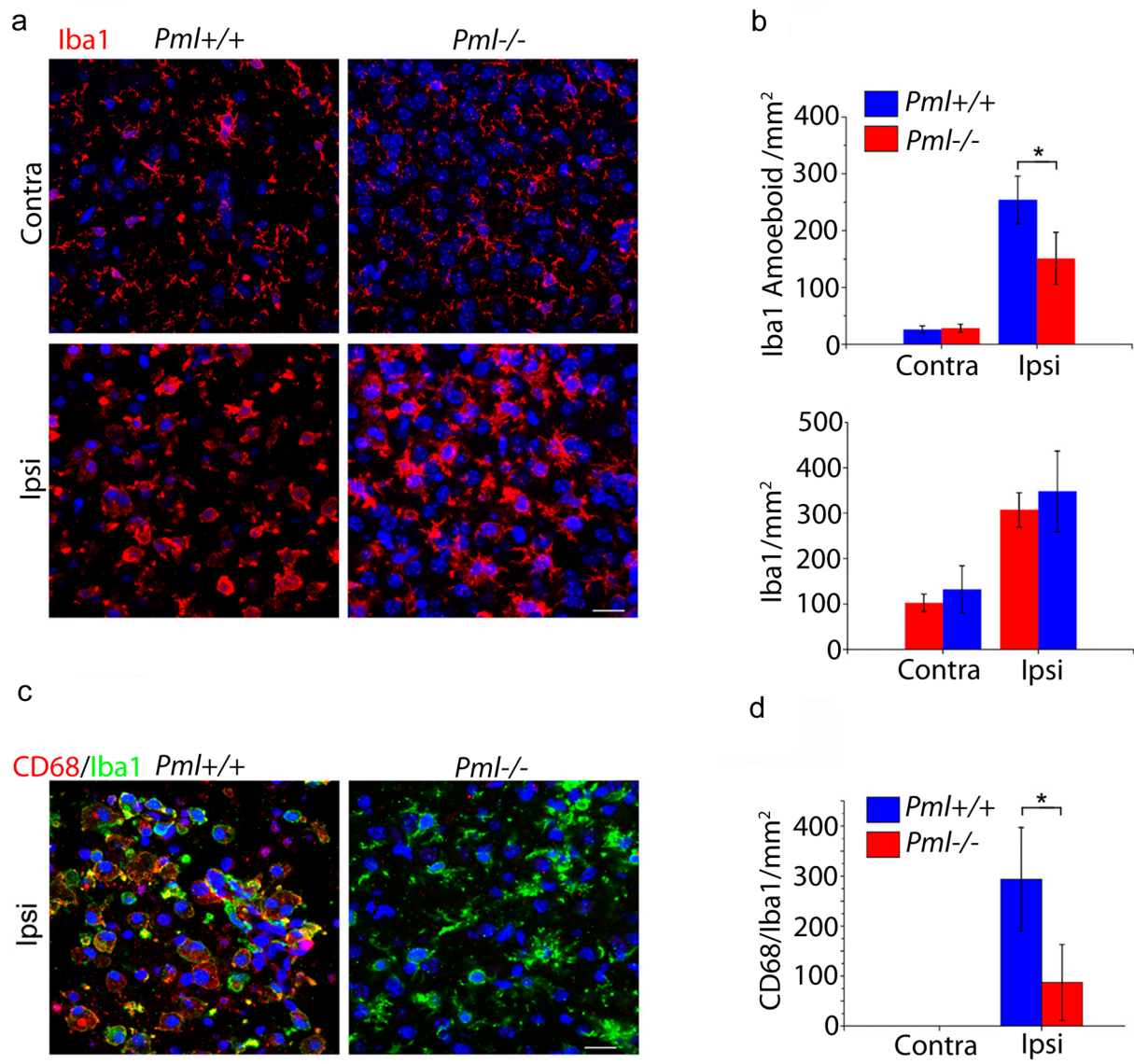

Supplement: Supplementary Figure S4 [file cddis2016223x5.pdf]

Supplementary figure S5

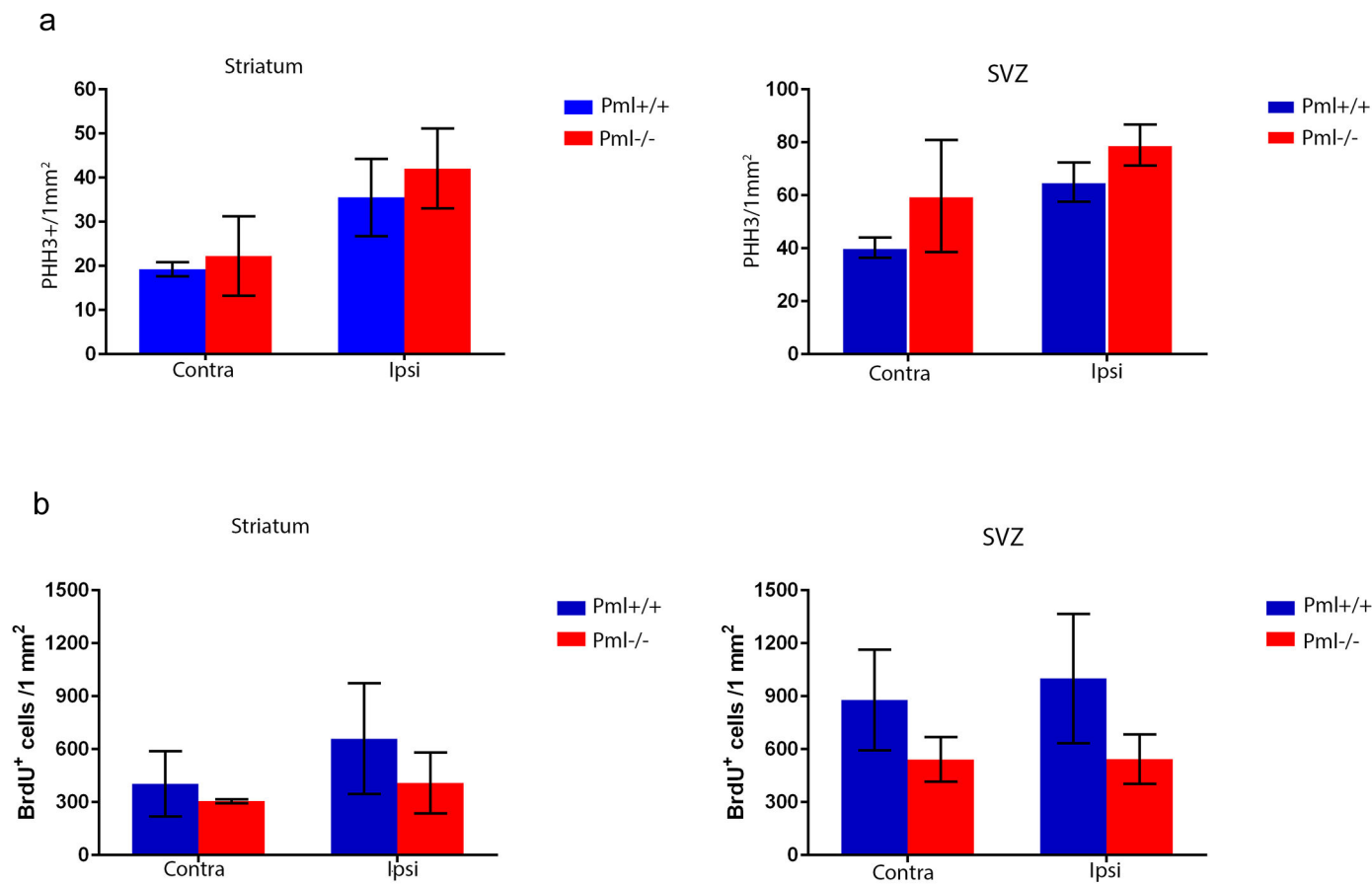

Supplement: Supplementary Figure S5 [file cddis2016223x6.pdf]

Supplementary figure S6

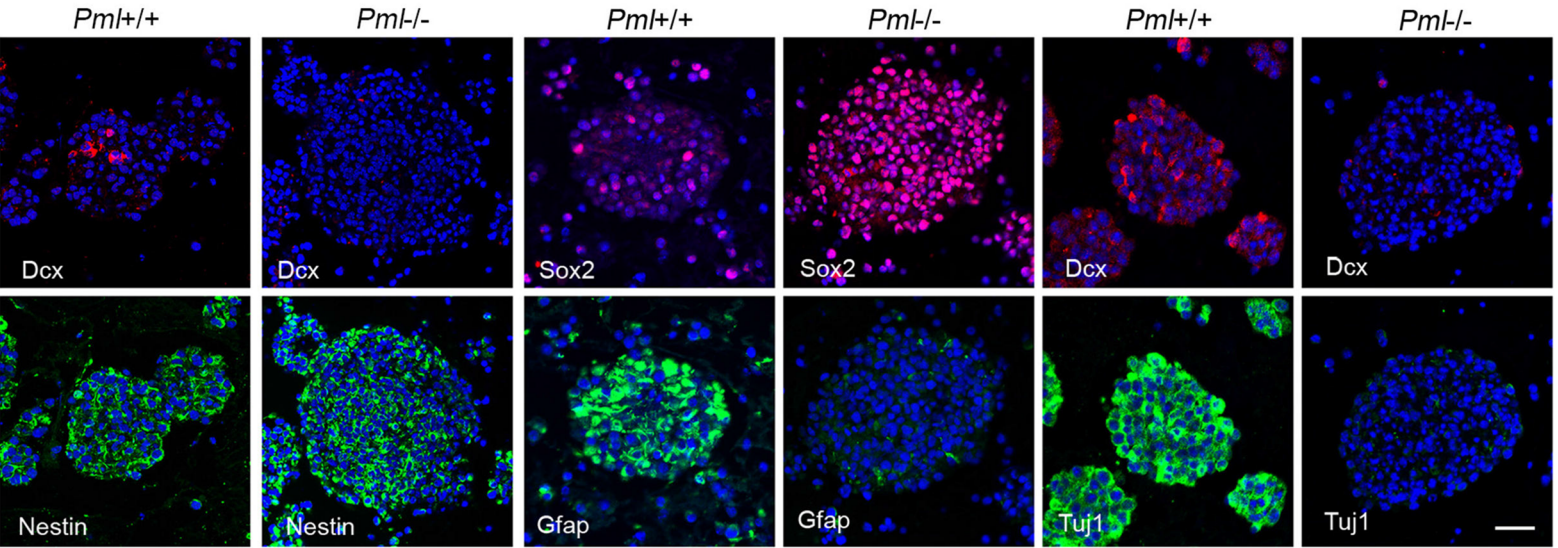

Supplement: Supplementary Figure S6 [file cddis2016223x7.pdf]
